# Stramonium in Pneumonia

**Published:** 1887-07

**Authors:** Julius G. Schmitt

**Affiliations:** Rochester, N. Y.


### STRAMONIUM IN PNEUMONIA.

Julius G. Schmitt, M. D., Rochester, N. Y.

The remedy is a rare one in this disease, and Hering in his *Materia Medica* only mentions it thus : "Pain in breast, cough, and other pure pneumonic symptoms during recovery from meningitis." Having had, however, two cases of pneumonia crouposa, where Stramonium proved curative, I thought them of sufficient interest to read their history to you, the more so, as these cases again confirm the law of Similia similibus curantur.

On March 20th, 1885, at four P. M., I saw Miss M. S., a young girl of nineteen years of age, of a delicate figure, with blonde hair and blue eyes. She hardly answers any questions, wants to be quiet. Left cheek is red and hot. Complains of pain in left side of chest and left hypochondrium, the latter painful to touch. No perceptible dullness in percussion over left lung, but auscultation reveals crepitant rales in lower left lung in axillary line, and there is enlargement of and dullness over spleen. She prefers to lie on her back. There is cough with pain in occiput and abdomen, with white mucous expectoration mixed with a little bright red blood. Occiput is so painful that she can hardly lie on it. Great thirst, pulse one hundred and forty-four, temperature one hundred and three and a half. She felt very bad at noon and almost fainted away. ℞ Sulphur$^{cm}$, one dose, dry on the tongue.

There was a little improvement the next day, but the day following she was worse again. Sulphur$^{cm}$ repeated did no good, neither did the following remedies: Belladonna$^{cm}$, Arsenic$^{cm}$, Verat. alb.$^{cm}$, Tartarus emeticus$^{6}$, Arnica$^{cm}$, Pulsatilla$^{cm}$, all of which were given according to the best of my ability. So the 26th of March had come, when I saw her at nine P. M., and the following symptoms were taken down:

Pulse, one hundred and thirty-two; temperature, 103.3. She had a pretty fair afternoon, but worse since seven P. M. Breathing very short and labored, with a continuous rattling on the chest, which was relieved by a profuse, slimy, watery expectoration, mixed with reddish purulent matter.

Auscultation showed large bubbling rales all over both lungs, and the percussion sound was flat over the entire back. Urine had not been voided since this morning, but there had been a loose alvine evacuation at one P. M. As soon as she falls into a doze, a profuse perspiration will break out all over her body. *Cough*

*increased by cold drinks,* fits of suffocation following the cough, with desire to have doors and windows open. *Craving for beer,* for no other nourishment. A new symptom was elicited that evening, which proved to be the keynote for the case, namely: *Great desire for light,* and if the room is dark, she thinks she will be smothered. Stramonium flashed at once through my brain, and looking up this remedy for further confirmation of its similitude, I found the craving for beer given a prominent place amongst desires and aversions in the little pamphlet of Guernsey, and in Lee's Repertory for Cough, the aggravation from cold drinks. Resting now on the famous three legs, I prescribed: ℞ Stramonium$^{cm}$, one dose dry on the tongue.

March 27th.—I must confess that I could hardly wait until I got to my patient that morning, and when I finally saw her at ten A. M., the first glance assured me that Homœopathy, applied according to Hahnemann, was going to register another of the many triumphs over disease. The pulse was one hundred and fourteen; temperature one hundred and one. Urine had been voided in large quantities. Rattling in chest had gone, breathing was much easier, expectoration had become tough and thick, so that it was hard to detach from the lips, there was very little reddish purulent matter with it. Rales on chest were finer, which indicated a mitigation of the œdematous condition of the previous evening (this is, by the by, the benefits I derive from pathology, no others). An hour after she received the dose, the mother told me she was greatly excited, thought she was going to die, talked out of her head, and wanted her mother constantly with her. These symptoms, however, subsided after a while, and she continued to feel easier all over. Continued Sac. lac.

Seven P. M., pulse, one hundred and four; temperature, 100.3. Cough harder, but not frequent. Expectoration very difficult. Breathing a little shorter again. This afternoon she saw faces (Stramonium). At four and a half P. M. she perspired profusely, and wanted to be covered. Third day. Sac. lac.

March 28th.—Ten and a-half A. M. She slept last night from eleven P. M. to five A. M. without hardly any cough. Expectoration now perfectly white, but still very tough. No movement from the bowels, no thirst. Pulse, ninety-four; temperature, ninety-nine. Sac. lac.

March 29th.—Eleven A. M. Temperature, one hundred; pulse, ninety. Talking of other people affects her very much, oppresses her chest. Her mother gave her a dose of Rochelle Salts because she had an unsuccessful desire to move her bowels.

"Mother got frightened at me," and daughter received ℞ Nux vomica$^{cm}$, one dose.

March 30th.—She feels much better, sits upright in bed, and enjoys her lunch. She slept all night. Bowels moved natural at four A. M. Expectoration is grayish; auscultation shows slight bronchial breathing in the middle of right lung; no respiratory murmur at all in both inferior lungs, where there is complete dullness on percussion. Pulse, one hundred and four; temperature, 99.3. ℞ Sac. lac.

March 31st.—Pulse, one hundred and twenty; temperature, one hundred and one. New symptoms were: Chill from seven to eight P. M. last night, and she wants to be covered now. Pains all over the chest. She cannot stir on account of them. Soles of feet hurt her, as if the skin had been pulled off. The least noise will disturb her sleep. She awakens frightened, sees her late sister, who died under "scientific" regular treatment four weeks ago. There is still the desire for beer and light. ℞ Stramonium$^{cm}$, one dose.

April 6th.—The action of the remedy, being not disturbed this time, went on in a steady improvement of the patient until to-day, when other symptoms came up calling for a dose of Sulphur$^{cm}$, which ended the sickness, so that she could be discharged on the 11th of April.

Case II.—November 20th, 1885, ten A. M.—Mr. Geo. W. H., thirty years old, blonde, robust, lives *in the same part* of the city where Case I was treated, has had right-sided pneumonia since the 16th, and had received Rhus tox. and then Belladonna without benefit. *Status presens:* Pulse, one hundred and two; temperature, one hundred and three. Very thirsty for cold water. Moaning and great loquacity, with high delirium; wants to jump out of bed; can hardly be kept there; talks about horses in a barn that he has to feed, etc.; *thinks one of his legs is cut off from his body; wants his wife to stay with him all the time and light in the room;* imagines he is going to die; countenance besotted; coughs considerably, and raises yellow and bloody mucus; complains of severe frontal headache, which had been a prominent symptom all along; high fever from four P. M. until five A. M.; no movement from bowels since the commencement of his sickness; urine clear, but dark red. There is now also bronchial breathing and dullness on percussion on lower left lung. ℞ Stramonium$^{cm}$, one dose dry on tongue.

Half-past five P. M.—Since three P. M. he is in a profuse perspiration, his delirium is less, he is brighter, and does not moan so much. Expectoration light yellow, not bloody any more. Pulse, one hundred and two; temperature, 102.2. Headache less; does not talk any more about his cut-off leg. ℞ Sac. lac.

November 21st, twelve M.—Found him sleeping; he has, however, been delirious through the night, but not so violent as the nights before. Pulse, ninety-four; temperature, 101.1. Breathing, thirty-six in a minute. Bronchial breathing in upper right lung; left lung only vesicular breathing. Expectoration yellowish green, tough, difficult to raise. Some headache yet. Thirst the same, but he is more inclined to take nourishment now. Bowels moved twice, thin, yellow. Urine clear, and of lighter color. There is still some delirium. ℞ Sac. lac.

November 22d.—He slept almost all of last night. There was some slight delirium, none this morning. Profuse warm perspiration since last night. Pulse, sixty-six; temperature, 99.2; breathing, twenty-six in a minute. Bowels have not moved again. Urine dark brown, but clear; expectoration greenish, thick, and again mixed with a little blood. No examination of the chest was made on account of the warm, profuse perspiration. ℞ Sac. lac.

November 23d.—Better. Pulse, sixty-six; temperature, 98.2; breathing, twenty. Bowels moved four times, thin and watery; no expectoration. Some fine rales on right lung. No bronchial breathing any more. ℞ Sac. lac.

November 25th.—No chest symptoms remain. Tongue is coated thick yellowish; there is an empty, gone feeling in stomach and bowels, and two to three thin stools early in the morning. ℞ Sulphur$^{30}$, one dose.

November 27th.—Discharged as cured.

